# Supplementary material for: Modeling glioblastoma heterogeneity as a dynamic network of cell states
Source: Mol Syst Biol. 2021 Sep 16;17(9):e10105. doi: 10.15252/msb.202010105 (PMC8444284; doi:10.15252/msb.202010105)
Supplement: Supplementary file 6 — Source Data for Figure 5 [file MSB-17-e10105-s004.zip › Figure5A_sourcedata/GSEA_3071/hallmarks_stateVI.GseaPreranked.1623742557031/HALLMARK_MYC_TARGETS_V1.html]

Details for gene set HALLMARK\_MYC\_TARGETS\_V1[GSEA]

|  || Dataset | state63071 |
| Phenotype | NoPhenotypeAvailable |
| Upregulated in class | na\_pos |
| GeneSet | HALLMARK\_MYC\_TARGETS\_V1 |
| Enrichment Score (ES) | 0.3188836 |
| Normalized Enrichment Score (NES) | 2.3148699 |
| Nominal p-value | 0.0 |
| FDR q-value | 0.0 |
| FWER p-Value | 0.0 |
Table: GSEA Results Summary

  

Fig 1: Enrichment plot: HALLMARK\_MYC\_TARGETS\_V1      
 Profile of the Running ES Score & Positions of GeneSet Members on the Rank Ordered List

  

| PROBE | GENE SYMBOL | GENE\_TITLE | RANK IN GENE LIST | RANK METRIC SCORE | RUNNING ES | CORE ENRICHMENT || 1 | CDC20 |  |  | 15 | 1.073 | 0.0213 | Yes |
| 2 | ODC1 |  |  | 38 | 0.918 | 0.0370 | Yes |
| 3 | MYC |  |  | 69 | 0.815 | 0.0479 | Yes |
| 4 | NPM1 |  |  | 114 | 0.717 | 0.0526 | Yes |
| 5 | EIF3D |  |  | 152 | 0.663 | 0.0579 | Yes |
| 6 | IMPDH2 |  |  | 188 | 0.609 | 0.0625 | Yes |
| 7 | LDHA |  |  | 197 | 0.604 | 0.0747 | Yes |
| 8 | RUVBL2 |  |  | 212 | 0.595 | 0.0849 | Yes |
| 9 | CCT5 |  |  | 225 | 0.585 | 0.0954 | Yes |
| 10 | MRPS18B |  |  | 262 | 0.558 | 0.0986 | Yes |
| 11 | CCT4 |  |  | 272 | 0.553 | 0.1092 | Yes |
| 12 | EIF2S1 |  |  | 287 | 0.547 | 0.1183 | Yes |
| 13 | SRM |  |  | 292 | 0.544 | 0.1301 | Yes |
| 14 | PWP1 |  |  | 319 | 0.528 | 0.1353 | Yes |
| 15 | EIF4A1 |  |  | 329 | 0.523 | 0.1453 | Yes |
| 16 | RSL1D1 |  |  | 336 | 0.521 | 0.1560 | Yes |
| 17 | NOP16 |  |  | 392 | 0.492 | 0.1522 | Yes |
| 18 | NME1 |  |  | 405 | 0.487 | 0.1604 | Yes |
| 19 | RACK1 |  |  | 407 | 0.486 | 0.1716 | Yes |
| 20 | EIF3B |  |  | 418 | 0.482 | 0.1803 | Yes |
| 21 | C1QBP |  |  | 435 | 0.476 | 0.1871 | Yes |
| 22 | CCT2 |  |  | 441 | 0.474 | 0.1970 | Yes |
| 23 | CCT7 |  |  | 460 | 0.467 | 0.2030 | Yes |
| 24 | PSMC4 |  |  | 472 | 0.463 | 0.2110 | Yes |
| 25 | DDX21 |  |  | 511 | 0.452 | 0.2110 | Yes |
| 26 | COX5A |  |  | 528 | 0.449 | 0.2172 | Yes |
| 27 | RPS5 |  |  | 534 | 0.446 | 0.2264 | Yes |
| 28 | RPL18 |  |  | 564 | 0.436 | 0.2285 | Yes |
| 29 | RPLP0 |  |  | 566 | 0.435 | 0.2386 | Yes |
| 30 | RPS6 |  |  | 593 | 0.427 | 0.2414 | Yes |
| 31 | PABPC4 |  |  | 633 | 0.415 | 0.2403 | Yes |
| 32 | PRDX4 |  |  | 647 | 0.412 | 0.2465 | Yes |
| 33 | FBL |  |  | 657 | 0.411 | 0.2537 | Yes |
| 34 | EIF3J |  |  | 664 | 0.408 | 0.2617 | Yes |
| 35 | RAN |  |  | 712 | 0.394 | 0.2578 | Yes |
| 36 | ETF1 |  |  | 715 | 0.393 | 0.2667 | Yes |
| 37 | TCP1 |  |  | 758 | 0.384 | 0.2639 | Yes |
| 38 | SLC25A3 |  |  | 828 | 0.372 | 0.2533 | Yes |
| 39 | SSBP1 |  |  | 838 | 0.370 | 0.2596 | Yes |
| 40 | RRP9 |  |  | 872 | 0.365 | 0.2590 | Yes |
| 41 | ABCE1 |  |  | 889 | 0.362 | 0.2631 | Yes |
| 42 | PHB |  |  | 918 | 0.357 | 0.2637 | Yes |
| 43 | PSMA7 |  |  | 930 | 0.356 | 0.2691 | Yes |
| 44 | ACP1 |  |  | 970 | 0.350 | 0.2664 | Yes |
| 45 | AIMP2 |  |  | 973 | 0.349 | 0.2741 | Yes |
| 46 | PA2G4 |  |  | 986 | 0.348 | 0.2790 | Yes |
| 47 | RPS2 |  |  | 990 | 0.348 | 0.2865 | Yes |
| 48 | CYC1 |  |  | 1042 | 0.338 | 0.2801 | Yes |
| 49 | UBE2L3 |  |  | 1076 | 0.333 | 0.2787 | Yes |
| 50 | IFRD1 |  |  | 1085 | 0.333 | 0.2844 | Yes |
| 51 | PGK1 |  |  | 1099 | 0.331 | 0.2886 | Yes |
| 52 | EIF4G2 |  |  | 1100 | 0.330 | 0.2965 | Yes |
| 53 | CCT3 |  |  | 1107 | 0.329 | 0.3026 | Yes |
| 54 | PSMA6 |  |  | 1117 | 0.327 | 0.3079 | Yes |
| 55 | SERBP1 |  |  | 1126 | 0.327 | 0.3134 | Yes |
| 56 | AP3S1 |  |  | 1135 | 0.326 | 0.3189 | Yes |
| 57 | PSMB2 |  |  | 1171 | 0.320 | 0.3166 | No |
| 58 | PSMB3 |  |  | 1210 | 0.314 | 0.3134 | No |
| 59 | MRPL23 |  |  | 1247 | 0.310 | 0.3106 | No |
| 60 | POLD2 |  |  | 1265 | 0.308 | 0.3131 | No |
| 61 | PSMA1 |  |  | 1276 | 0.306 | 0.3176 | No |
| 62 | VDAC1 |  |  | 1362 | 0.296 | 0.3006 | No |
| 63 | MRPL9 |  |  | 1365 | 0.296 | 0.3071 | No |
| 64 | CSTF2 |  |  | 1456 | 0.284 | 0.2885 | No |
| 65 | FAM120A |  |  | 1469 | 0.283 | 0.2918 | No |
| 66 | HSP90AB1 |  |  | 1492 | 0.281 | 0.2923 | No |
| 67 | HDGF |  |  | 1502 | 0.279 | 0.2964 | No |
| 68 | PSMD7 |  |  | 1518 | 0.277 | 0.2988 | No |
| 69 | NOP56 |  |  | 1531 | 0.276 | 0.3020 | No |
| 70 | KPNB1 |  |  | 1538 | 0.275 | 0.3068 | No |
| 71 | HSPD1 |  |  | 1556 | 0.274 | 0.3085 | No |
| 72 | RAD23B |  |  | 1586 | 0.270 | 0.3068 | No |
| 73 | EIF4H |  |  | 1635 | 0.265 | 0.2996 | No |
| 74 | NHP2 |  |  | 1656 | 0.264 | 0.3002 | No |
| 75 | EIF1AX |  |  | 1657 | 0.263 | 0.3065 | No |
| 76 | PSMD3 |  |  | 1688 | 0.261 | 0.3042 | No |
| 77 | NOLC1 |  |  | 1699 | 0.260 | 0.3076 | No |
| 78 | PPIA |  |  | 1715 | 0.259 | 0.3095 | No |
| 79 | SNRPA1 |  |  | 1720 | 0.259 | 0.3145 | No |
| 80 | RNPS1 |  |  | 1822 | -0.252 | 0.2920 | No |
| 81 | HNRNPA3 |  |  | 1914 | -0.266 | 0.2727 | No |
| 82 | CBX3 |  |  | 1983 | -0.275 | 0.2600 | No |
| 83 | NCBP2 |  |  | 2049 | -0.286 | 0.2485 | No |
| 84 | TFDP1 |  |  | 2143 | -0.304 | 0.2294 | No |
| 85 | RFC4 |  |  | 2185 | -0.312 | 0.2253 | No |
| 86 | MCM5 |  |  | 2424 | -0.359 | 0.1666 | No |
| 87 | SMARCC1 |  |  | 2522 | -0.383 | 0.1484 | No |
| 88 | CDK2 |  |  | 2746 | -0.439 | 0.0958 | No |
| 89 | CDC45 |  |  | 2776 | -0.448 | 0.0983 | No |
| 90 | MCM7 |  |  | 2808 | -0.460 | 0.1005 | No |
| 91 | MCM6 |  |  | 2834 | -0.468 | 0.1046 | No |
| 92 | RRM1 |  |  | 3070 | -0.574 | 0.0519 | No |
| 93 | DEK |  |  | 3098 | -0.583 | 0.0581 | No |
| 94 | USP1 |  |  | 3233 | -0.699 | 0.0370 | No |
| 95 | MCM4 |  |  | 3254 | -0.715 | 0.0483 | No |
| 96 | DUT |  |  | 3307 | -0.769 | 0.0520 | No |
| 97 | TYMS |  |  | 3333 | -0.798 | 0.0639 | No |
| 98 | PCNA |  |  | 3415 | -0.922 | 0.0630 | No |
Table: GSEA details [plain text format]

  

Fig 2: HALLMARK\_MYC\_TARGETS\_V1: Random ES distribution      
 Gene set null distribution of ES for **HALLMARK\_MYC\_TARGETS\_V1**
